# Supplementary material for: Efficacy and Tolerability of Perampanel in Brain Tumor-Related Epilepsy: A Systematic Review
Source: Biomedicines. 2023 Feb 21;11(3):651. doi: 10.3390/biomedicines11030651 (PMC10045654; doi:10.3390/biomedicines11030651)
Supplement: Supplementary file 1 [file biomedicines-11-00651-s001.zip › biomedicines-2203038-supplementary.pdf]

Table S1: Search domain and search terms (Cochrane/Web of Science/Scopus/ClinicalTrial.gov/Who Trial Registry).

Database	MedLine/Pubmed	Embase	Cochrane
	(("perampanel" [Supplementary Concept] OR Perampanel)) AND (("Epilepsy"[Mesh] OR epilep*[title/abstract] OR "Seizures"[Mesh] OR seizure*[title/abstract]) AND ("Glioma"[Mesh] OR glioma*[title/abstract] OR brain tumor[title/abstract] OR brain tumour[title/abstract] OR brain cancer[title/abstract] OR brain neoplasm[title/abstract] OR brain metastas*[title/abstract] OR "Meningioma"[Mesh] OR meningioma*[title/abstract]))	('brain cancer'/exp OR 'brain cancer' OR 'brain tumor' OR 'brain tumour' OR 'brain neoplasm' OR 'glioma'/exp OR glioma OR 'brain metastas* ' OR 'meningioma'/exp OR 'meningioma') AND ('epilepsy'/exp OR epilep*:ab,ti OR 'seizure'/exp OR seizure*:ab,ti) AND ('perampanel'/exp OR perampanel)	perampanel in Title Abstract Keyword AND epilep* OR seizure* in Title Abstract Keyword AND brain cancer OR brain tumor OR brain neoplasm OR glioma OR brain metastas* OR meningioma in Title Abstract Keyword - (Word variations have been searched)
**Database**	**Scopus**	**Web of Science**	
	(TITLE-ABS-KEY (perampanel) AND TITLE-ABS-KEY (brain AND cancer OR brain AND tumor OR brain AND neoplasm OR glioma OR brain AND metastas* OR meningioma) AND TITLE-ABS-KEY (epilep* OR seizure*))	perampanel (All Fields) and brain cancer OR brain tumor OR brain neoplasm OR glioma OR brain metastas* OR meningioma (All Fields) and epilep* OR seizure* (All Fields)	
	**Clinical Trials**		
	**ClinicalTrial.gov**	**Who Trial Registry**	
	perampanel AND (brain cancer OR brain tumor OR brain neoplasm OR glioma)	perampanel AND (brain cancer OR brain tumor OR brain neoplasm OR glioma)	
